# A global climate niche for giant trees

**DOI:** 10.1111/gcb.14167

**Published:** 2018-05-02

**Authors:** Marten Scheffer, Chi Xu, Stijn Hantson, Milena Holmgren, Sietse O. Los, Egbert H. van Nes

**Affiliations:** ^1^ Department of Aquatic Ecology and Water Quality Management Wageningen University Wageningen The Netherlands; ^2^ School of Life Sciences Nanjing University Nanjing China; ^3^ Karlsruhe Institute of Technology Institute of Meteorology and Climate research Atmospheric Environmental Research Garmisch‐Partenkirchen Germany; ^4^ Resource Ecology Group Wageningen University Wageningen The Netherlands; ^5^ Department of Geography Swansea University Swansea UK

**Keywords:** alternative ecosystem state, canopy height, LiDAR, precipitation temperate rainforest, remote sensing, resilience, threshold, tropical rainforest

## Abstract

Rainforests are among the most charismatic as well as the most endangered ecosystems of the world. However, although the effects of climate change on tropical forests resilience is a focus of intense research, the conditions for their equally impressive temperate counterparts remain poorly understood, and it remains unclear whether tropical and temperate rainforests have fundamental similarities or not. Here we use new global data from high precision laser altimetry equipment on satellites to reveal for the first time that across climate zones ‘giant forests’ are a distinct and universal phenomenon, reflected in a separate mode of canopy height (~40 m) worldwide. Occurrence of these giant forests (cutoff height > 25 m) is negatively correlated with variability in rainfall and temperature. We also demonstrate that their distribution is sharply limited to situations with a mean annual precipitation above a threshold of 1,500 mm that is surprisingly universal across tropical and temperate climates. The total area with such precipitation levels is projected to increase by ~4 million km^2^ globally. Our results thus imply that strategic management could in principle facilitate the expansion of giant forests, securing critically endangered biodiversity as well as carbon storage in selected regions.

## 1 INTRODUCTION

Tropical and temperate rainforest provide important ecosystem services to humanity (Millennium Ecosystem Assessment, 2005). Provisioning (e.g., wood) and regulating (e.g., climate) services can more easily be quantified than cultural and spiritual services. Yet, it could be argued that the latter are equally important to humanity because our choices and attitudes are strongly influenced by nonrational drivers, as demonstrated for instance by the work of Nobel laureates Kahneman and Thaler (Kahneman, 2011). While spiritual ecosystem services are hard to measure, the gigantic trees of tropical and temperate rainforests clearly have a profound effect on human perception of nature. This is illustrated vividly by a quote from Darwin who, in the midst of the Brazilian forest, wrote “It is not possible to give an adequate idea of the higher feelings of wonder, admiration, and devotion which fill and elevate the mind.” (Darwin, 1958). Similarly, John Steinbeck noted that in the temperate rainforest “The redwoods, once seen, leave a mark or create a vision that stays with you always…. From them comes silence and awe” (Steinbeck, 1962).

Here we address the question whether these iconic forests are simply part of a continuum of forest types or rather a real class apart when it comes to their height resulting in a distinct mode in the frequency distribution of canopy height on a global scale. We also ask if it is possible to define the critical conditions for such giants to thrive. Until recently these questions were surprisingly hard to address, as systematic global measurement of canopy height was beyond our reach. This has changed with the availability of high precision laser altimetry equipment on satellites. We can now assess height distribution on a scale of 0.5° grid cells. In each grid cell, there is a large set of measurements based on laser beams sampling areas with a diameter of ~50–150 m. After correcting for topography, these measurements provide an estimate of the distribution of vegetation height (Los et al., 2012; Rosette, North, & Suárez, 2008). Here we explore this fascinating new source of information to assess for the first time whether giant forests are a distinct phenomenon and to determine the critical climatic conditions for their persistence.

## 2 MATERIALS AND METHODS

Rather than studying the height distribution of individual trees within forests, we focus on the highest parts of the canopies. We did this by determining the 90th percentile of vegetation height within each 0.5° grid cell (a conservative measure for the maximum canopy height). We excluded human‐dominated lands from analysis.

The global vegetation height product (downloaded from https://www.researchgate.net/publication/265641148_GLAS_height) was derived from the Geoscience Laser Altimeter System (GLAS) aboard the Ice, Cloud and land Elevation Satellite (ICESat) (Los et al., 2012). GLAS collected laser altimeter data intermittently between 20 February 2003 and 11 October 2009. Measurement campaigns were carried out one to three times a year and lasted from about a week to longer than a month. The GLAS along‐track sampling rate was around 172 m and footprints sizes varied between about 149 m to 51 m in diameter (major axis). Overall a few percent of the total land cover is covered by the measurements. Filters to identify spurious data were developed for a desert site; spurious data include data affected by clouds, atmosphere, or steep terrain (Los et al., 2012). Vegetation height was estimated from the elevation of the first return and the last two Gaussians using the elevation of the Gaussian with the largest amplitude (Los et al., 2012). Based on the desert analysis, a minor adjustment was applied to the heights from the model by Rosette et al. (2008). The filtered and adjusted heights were subsequently tested on aircraft vegetation height data from ten sites across the globe (Canada, the Netherlands, Sweden, United Kingdom, Peru, Germany, and Australia). These sites differed in relief, vegetation density, tree age, and tree height. The filter improved the correspondence between aircraft and satellite estimated vegetation height, the correlation coefficient increased from 0.33 to 0.76, and the root means square error decreased by a factor 3 to about 4.5–6 m. More stringent filtering (difference aggressive filter and weaker filter) increased the 90th percentile in tropical forest by 0–3 m, whereas in mountainous regions outside the tropics height decreased by 0–4 m. No differences were found in height estimates across GLAS laser campaigns (Los et al., 2012). Finally, a histogram of heights from 0 to 70 m in 0.5 m intervals were aggregated in each 0.5 × 0.5° grid cell (within each cell, the numbers of effective LiDAR footprints are 1,444 ± 1,297 and 4,247 ± 2,968 for the filtered and unfiltered data, respectively), and the 90th percentile highest heights were used in our analysis. This provides direct estimates of vegetation heights that do not involve climatic variables or vegetation cover, thus allowing for correlative inference of these variables with height.

**Figure 1:**
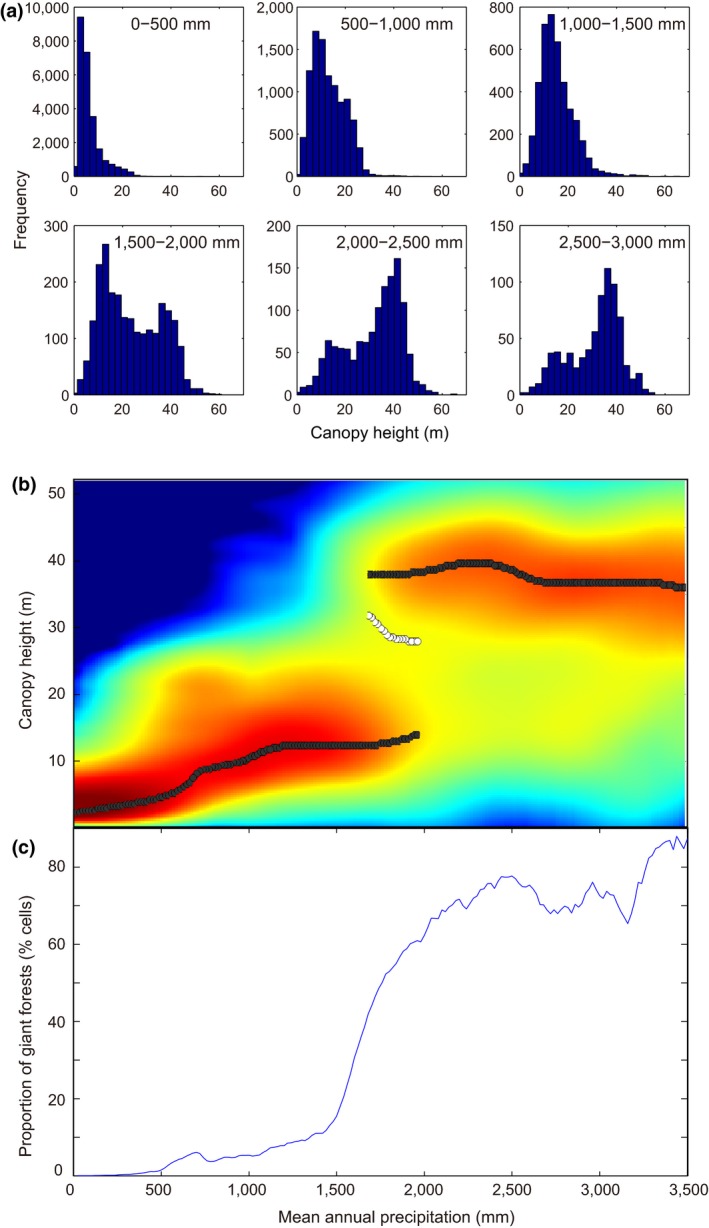
The maximum canopy height of the world's forests as a function of mean annual precipitation. (a) Frequency distributions of canopy height for different ranges of precipitation illustrating that a distinct mode with a height of ~40 m arises for precipitation >1,500 mm/year. (b) Maxima (filled dots) in the probability density (the blue‐yellow‐red scale represents a point density gradient from low to high) of canopy heights illustrate the distinct character of giant forest, and minima (open dots) illustrate that a canopy height of ~25 m is relatively rare. (c) The percentage of 0.5° grid cells that has giant forest (canopy height > 25 m) rises steeply from an annual precipitation of 1,500 mm onwards (see materials and methods in Supporting information)

Before analyses, we excluded human‐used, water, and bare areas using the Globcover dataset during 2004–2006 by European Space agency (Defourny et al., 2006)(downloaded from http://due.esrin.esa.int/page_globcover.php). This remote‐sensing product based on the MERIS instrument aboard ENVISAT provides information on 22 categories of global land cover at 300 m resolution. Tree cover data were extracted from the MODIS Vegetation Continuous Field (VCF) Collection 5 dataset for the year 2001 (downloaded from http://www.landcover.org/data/vcf/). The MODIS VCF product estimates percent tree cover at a spatial resolution of 250 m. The mean annual precipitation (MAP) data at 1 km resolution were downloaded from the WorldClim website (Hijmans, Cameron, Parra, Jones, & Jarvis, 2005)(http://worldclim.org/). The mean annual water balance (WB) data from the GNV 183 dataset at 0.5° resolution (http://geonetwork.grid.unep.ch/) were used to estimate net precipitation. The WB estimates were based on monthly averages of climate data during 1920–1980 (Tateishi & Ahn, 1996). To check the precipitation condition on which giant forests are dependent, we computed the stability landscape directly from the data (Livina, Kwasniok, & Lenton, 2010). We estimated the equilibrium values by determining the local minima and maxima of the probability density function numerically (Figure 1b); we also plotted the percentage of giant forest cells (i.e., cells with canopy taller than 25 m) within a window of 200 mm MAP moving along the MAP gradient at a step of 20 mm (Figure 1c). We further investigated the distribution of giant forests by climatic regions (i.e., tropics vs. nontropics) using the FAO map of thermal climate zones of the world (http://gaez.fao.org/). Before analyses, all datasets were resampled to a consistent spatial resolution of 0.5 × 0.5°.

To test if our results are dependent of precipitation data used, and if the use of filter in the height product can have major influences, we repeated our analyses using the mean annual precipitation data during 1950–2000 from the Climate Research Unit (CRU, Harris, Jones, Osborn, & Lister, 2014, downloaded from http://www.cru.uea.ac.uk/data) and the raw (unfiltered) height data. In addition, to assess if forest harvest could be responsible for the observed pattern, we repeated our analyses excluding areas with significant recent forest loss. Using Landsat images, Hansen et al. (Hansen et al., 2013) compiled a global forest loss data during 2000–2013 at a fine resolution of 30 m. We aggregated this dataset to 0.5‐degree grid cells and excluded the cells with forest loss >0.5% of the grid cell area.

## 3 RESULTS AND DISCUSSION

**Figure 2:**
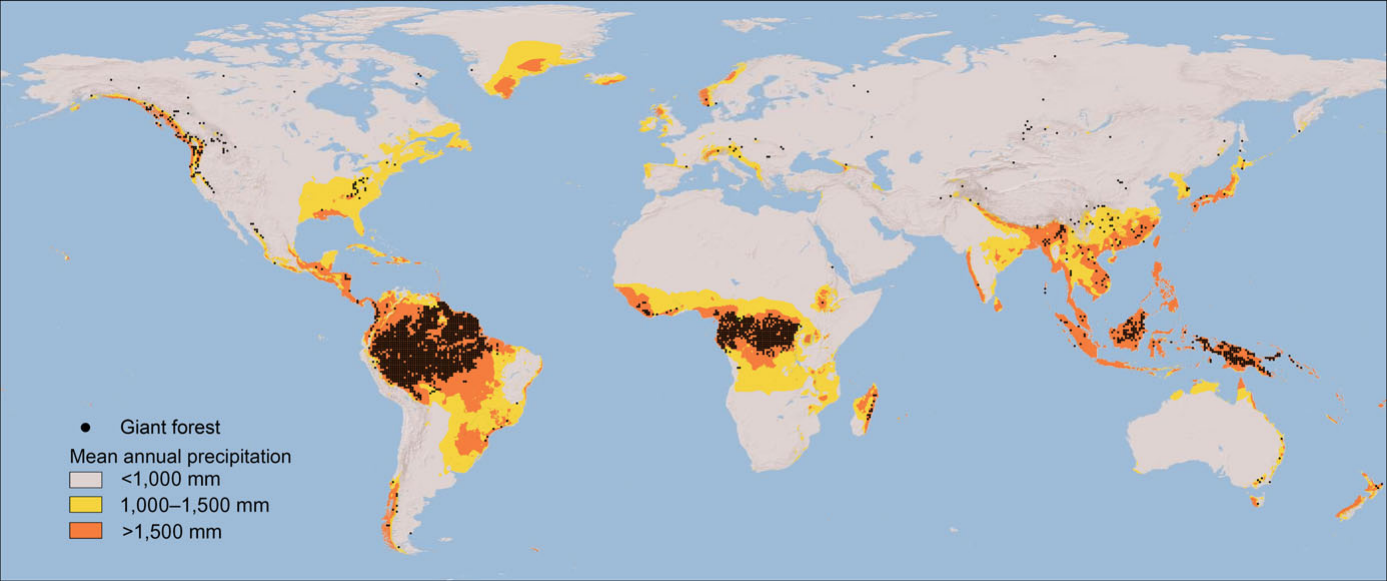
Global distribution of giant forests (canopy > 25 m) in relation to mean annual precipitation

As a first step, we examine the frequency distributions of the maximum canopy height at different rainfall levels worldwide (Figure 1). Rather than a gradual increase of tree height with precipitation (Klein, Randin, & Körner, 2015), our analysis reveals a marked discontinuity in canopy height distributions around a mean annual precipitation of ~1,500 mm. Below this critical precipitation, maximum canopy height peaks around a mode of 10–20 m. Although beyond a precipitation of 1,500 mm/year an alternative mode of tall forests arises with a maximum canopy height of ~40 m. Obviously, individual trees can be much taller than the ~40 m mode (Koch, Stillet, Jennings, & Davis, 2004). However, rather than looking at individual giants we focus here on the distinct tall mode that emerges from the global data. As individual giant trees are typically found within the tall canopies we detect, we will loosely refer to our tall mode (>25 m) as ‘giant forests’ hereafter. A map of the global distribution of such giant forests (Figure 2) confirms that they are typical of the global hotspots of high rainfall.

**Figure 3:**
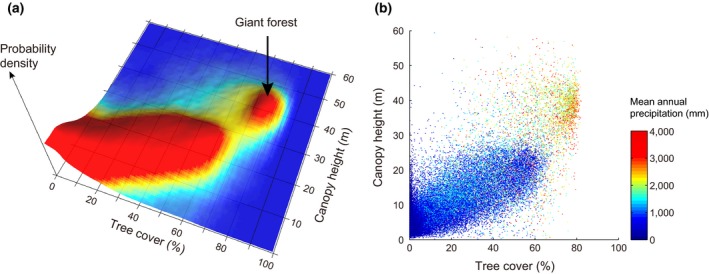
Distribution of canopy height as a function of tree cover illustrates that giant forest is a distinct phenomenon. (a) Probability density distribution of points as a function of tree cover and canopy height. (b) Color coding for the mean annual precipitation level illustrating that giant forest does not occur at rainfall below 1,500 mm/year (blue) and also that higher rainfall (yellow‐red) is no guarantee for giant forest

The idea that the giant forest is a qualitatively distinct phenomenon is supported by a marked discontinuity in the relationship between tree cover and tallness (Figure 3). Canopy height increases almost linearly with tree cover, but there is an abrupt break in this pattern at a cover of about 60%. Beyond that point, we find the giant forest with a tree cover of ~80% and a canopy height of ~40 m. Between the giant forest and the other forests of the world, there is a paucity in the sense that there are few forests with a cover around 60% and a canopy height around 25 m. To probe robustness of our findings against potential confounding effects of data preprocessing, we also repeated our analysis using a different precipitation database and unfiltered data on canopy height (Figures S1 and S2 respectively).

**Figure 4:**
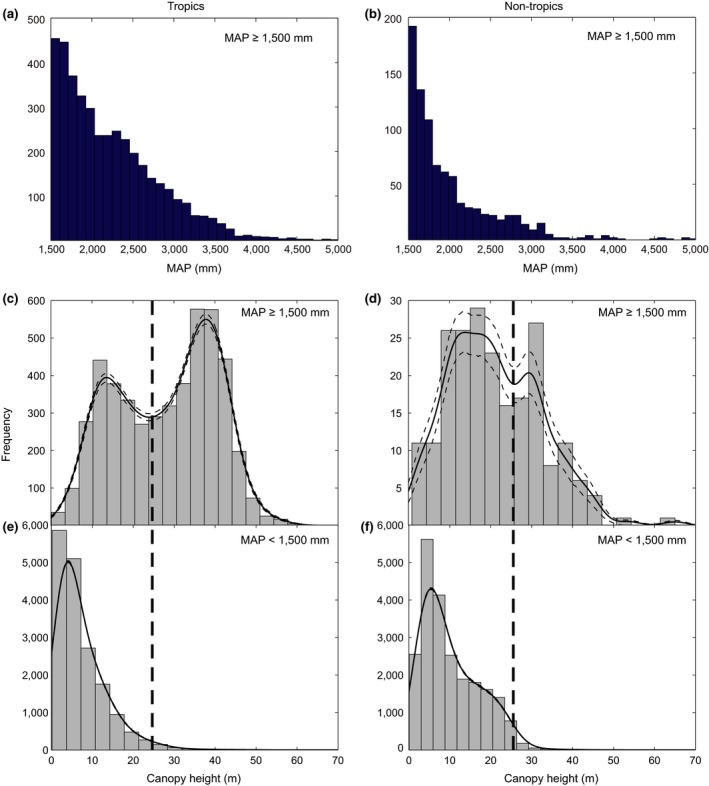
Distributions of mean annual precipitation and maximum canopy height in the tropics vs. the nontropics. Mean annual precipitation (panels a and b) in wet regions (MAP > 1,500 mm) is smoothly distributed, implying that it cannot explain the bimodality of canopy heights (panels c and d). The distinct mode of giant forest (>25 m) is absent in regions where mean annual precipitation (MAP) is below 1,500 mm (panels e and f). Note the different scales on the axis implying that that such very wet places are much rarer in the nontropics (panel b) than in the tropics (panel a). The probability density distributions (solid curves, with dashed curved representing the 95% confidence intervals) based on kernel density estimations are calculated using the *ksdensity* function in Matlab R2011b

Our results first of all raise the question what might explain the bimodality, or—phrased otherwise—the paucity of intermediate sized trees. As shown in a recent study (Van Nes et al., 2016; Xu et al., 2016) for the tropics, the discontinuity in canopy height corresponds to the sharp distinction between forests and savannas as alternative stable states (Hirota, Holmgren, Van Nes, & Scheffer, 2011; Staver, Archibald, & Levin, 2011). However, the giant forest also occurs in temperate climates, and patterns turn out to be surprisingly universal across climate zones. Both in tropical and in temperate climates, giant forest represents a distinct mode in canopy height distributions, and in both climate zones, canopies taller than 25 m require an annual precipitation beyond 1,500 mm (Figure 4). One possible explanation for bimodality of ecosystem states is bimodality of environmental conditions (Scheffer & Carpenter, 2003). This possibility cannot be entirely excluded as we do not have information about all environmental factors. However, the two modes cannot be explained from a bimodality of rainfall as mean annual precipitation is rather continuously distributed (Figure 4 panels a and b). Another possibility would be that the paucity of canopies of intermediate height would be due to a systematic global pattern in harvest. However, this seems unlikely. Although we cannot exclude a possible role of historical harvest patterns, we excluded human‐used lands, and also checked that the results are robust when only considering pixels without signs of recent forest loss (Figure S3).

Floristic observations support the idea that giant forests are a class apart. This is perhaps clearest in the tropics where savannas have a taxonomically distinct set of tree species that are fire adapted and smaller than forest species (Hoffmann & Franco, 2003). In temperate regions, the distinction may seem less obvious. However, the distribution of giant forests we detect in temperate regions corresponds largely (but not completely) to the distribution of so‐called temperate rainforests classified on the basis of floristic and structural characteristics observed on the ground (Alaback, 1991; Dellasala, 2011). One could think that a particular set of species is responsible for shaping the giant forests. However, the reverse may be true too. Indeed, as G. Evelyn Hutchinson framed it, the ‘evolutionary play’ takes place in an ‘ecological theater’. Only under special conditions can such tall trees evolve. On a similar somewhat philosophical level, tallness can be viewed as reflecting a tragedy of the commons resulting from a prisoners dilemma (Falster & Westoby, 2003). Trees must grow at least as tall as their neighbors to reach the light. Whatever the precise interplay of evolutionary and ecological mechanisms may be, the remotely sensed patterns we reveal are consistent with the idea (Alaback, 1991; Dellasala, 2011; Hoffmann, Orthen, & Vargas Do Nascimento, 2003) that in temperate as well as tropical climate zones giant forests are a distinct phenomenon rather than part of a continuum.

This brings us to the question what might explain our second key result, namely that giant forests across climate zones require a minimum of 1,500 mm mean annual precipitation. This seems unlikely to be an artifact of our novel data source. For instance, a classical floristic study independently suggests a similar limit of 1,400 mm mean annual precipitation for temperate rainforests (Alaback, 1991), and a study based on tree cover sets the limit for tropical rainforest to 1,500 mm (Hirota et al., 2011). Of course, mean annual precipitation is a rather crude indicator of climate. A more detailed analysis shows that in addition to the dominant effect of mean annual precipitation, there is also a marked effect of climate variability (Table S1). The occurrence of giant forest is negatively related to interannual variability of rainfall in all climate zones, and to seasonality of rainfall in the tropics. Also, seasonal low and high temperatures are negatively associated to the chances for giant forests in temperate regions. Effects of high temperatures are likely due to elevated vapor pressure deficits associated to tree mortality events (Allen et al., 2010), while negative effects of low temperatures may be associated to cavitation associated to freeze–thaw cycles (Willson & Jackson, 2006). Alternatively, deviations from an energetic optimum of ~13°C could play a role (Larjavaara, 2014).

Although climatic variability is an obvious qualifier for the emerging universality of the 1,500 mm precipitation requirement, it remains puzzling why roughly the same precipitation level would be critical for giant forests across climate zones. Rainforests in temperate and tropical regions differ widely. For instance, temperate rainforests harbor about two orders of magnitude less tree species than their tropical counterparts (Alaback, 1991). Also, among temperate forests, species composition as well as fire regimes differ widely between hemispheres (Alaback, 1991). Most importantly, (potential) evapotranspiration is obviously higher in warmer climates.

**Figure 5:**
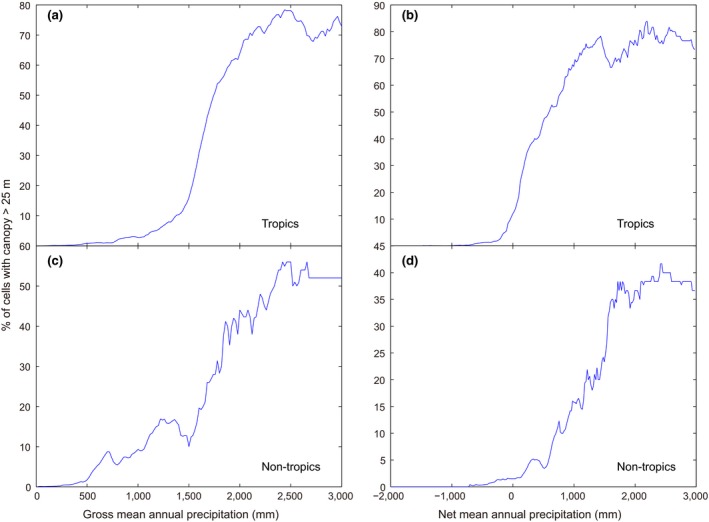
Probability of finding canopy taller than 25 m in the tropics (panels a and b) and the nontropics (panels c and d) as a function of *gross* (left) and *net* mean annual precipitation (mean annual precipitation minus potential evapotranspiration). For calculation of the percentage of giant forest cells within a window moving along the precipitation gradient at a step of 20 mm, adaptive window sizes (starting from 200 mm precipitation, and inflated to contain 50 data points at minimal) are used to avoid data scarcity at high precipitation levels

Many lines of evidence support the idea that water availability must be a key factor limiting giant growth. For instance, during natural droughts and in throughfall displacement experiments, the largest trees suffer the highest mortality upon drought (Nepstad, Tohver, David, Moutinho, & Cardinot, 2007), and physiological research suggests that leaf water stress due to gravity and xylem path length resistance are likely to ultimately limit tree height (Koch et al., 2004). However, availability of soil water depends on precipitation as well as evapotranspiration, which will differ markedly between temperate and tropical forests. Plotting the probability of finding giant forest against *net* precipitation suggests that precipitation exceeding evapotranspiration might approximate the critical condition in the tropics (Figure 5 panel b). However, puzzlingly, for temperate giant forest, the required net precipitation appears to be higher than in the tropics (Figure 5 panel d vs. b).

**Figure 6:**
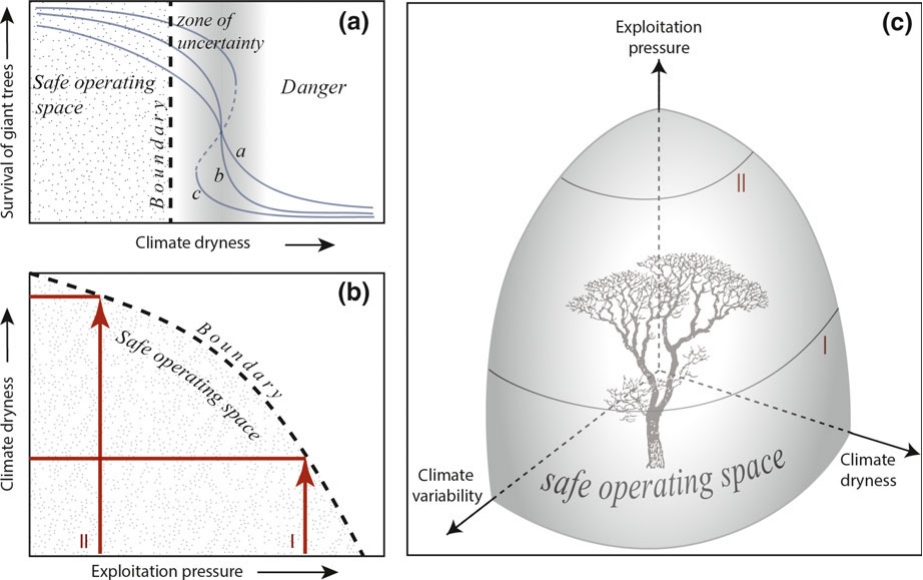
The concept of a safe operating space (Scheffer et al., 2015) applied to giant forests. (a) Possible responses of the survival of giant trees to decline in mean annual precipitation. The uncertainty in the shape and in the threshold level is accounted for by taking the boundary of the safe operating space at some distance from the best estimate of where the threshold is. (b) The safe boundary for precipitation can become lower if the forest canopy is opened as a result of exploitation. This implies that a drier climate can be tolerated by forest under low exploitation pressure allowing a well closed canopy (II) as compared to a more open forest resulting from higher exploitation pressure (I). (c) Climate variability is another important stressor that reduces the safe operating space for giant trees. (adapted from (Green et al., 2017))

A possible explanation is that additional local stressors reduce the ‘safe operating space’ (Scheffer et al., 2015) when it comes to the critical moisture for giant trees outside the tropics. The concept of a safe operating space stresses the fact that resilience of ecosystems always depends on a combination of multiple interacting stressors (Figure 6). Typically, increase in one stressor (e.g., thinning forest) can reduce the critical level tolerated for another stressor (e.g., drought) (Scheffer et al., 2015). Temperate giant forests have historically been under higher logging pressure than tropical forests. For instance, the range of Californian coastal redwoods has shrunk by 95% over the past 150 years (Koch et al., 2004). It might well be that the remaining pockets of giant trees are preferentially in the wettest places as resilience is largest there (Figure 6b red arrow I as opposed to II). Analyzing microscale distributional patterns could help to further resolve the role of local factors. For instance, in the southern end of the range redwoods are mostly restricted small pockets sheltered in narrow valleys (Lorimer et al., 2009). Opening forests makes them more vulnerable to water stress and fire (Malhi et al., 2008), as well as windfall (Lorimer et al., 2009), and recovery and survival of these forests might consequently be limited to the wettest, sheltered, and most productive places. Indeed, such preferential survival of forests in the face of human disturbance has been documented for oceanic islands, where despite similar human occupational history, islands that have more rain and more fertile soils kept their forests longer (Rolett & Diamond, 2004).

This view of a multivariate safe operating space has important corollaries. On one hand, tropical forests of the world might become limited to a much narrower band if human exploitation pressures reach those that have historically reduced the temperate rainforests. In addition to deforestation, road construction and fragmentation are major threats to tropical forest as they imply desiccation at forest edges which may boost tree mortality and fire risks during periods of drought (Malhi et al., 2008). If our inference is correct, this ongoing process could lead to a systematic retreat of tropical forest to regions where precipitation is well above the current 1,500 mm/year limit. On the other hand, the potential for giant forest across the globe might be much larger than suggested by its current distribution, and giant forests could potentially expand significantly if we enlarge the tolerated climate conditions by reducing exploitation pressure. Indeed, such management of the safe operating space (Scheffer et al., 2015) for giant forest may have potential. Although drought and heat events impose a risk to forests (Allen et al., 2010), many of the currently wet areas around the globe might well become wetter under future conditions, and the total area with annual precipitation levels beyond the critical 1,500 mm is projected to increase by ~4 million km^2^ globally both at the RCP 4.5 and the RCP 8.5 scenario (Figure S4). For comparison, that is roughly eight times the surface area of Spain. Clearly, it would be naïve to expect forest expansion to simply follow expected rainfall patterns. Projections of increased precipitation are uncertain (Greve et al., 2014) and climate extremes (Holmgren, Hirota, Van Nes, & Scheffer, 2013; Reichstein et al., 2013) and warmer conditions (Feeley, Joseph Wright, Nur Supardi, Kassim, & Davies, 2007) may negatively affect the potential for forests. Most importantly, political and economic barriers to a reallocation of land to the expansion of the world's cathedral forests are obviously formidable. On the other hand, although the growth of giant trees is slow, the gains are potentially large. In addition to carbon storage, expanded giant forests could secure much endangered biodiversity. Recent evidence suggests that even setting aside about one‐third of the land for forest in a landscape, may already secure most of the associated biodiversity (Banks‐Leite et al., 2014) due to a surprisingly generic threshold of about 30% forest cover for species richness in fragmented landscapes (Andrén, 1994; Banks‐Leite et al., 2014).

In conclusion, our analysis reveals that giant forest is a distinct phenomenon worldwide, which—depending on human disturbance—occurs beyond a sharply defined universal rainfall level. Understanding the interactive effects of climate and disturbance better may help us preserve and perhaps expand the range of these iconic ecosystems, harboring much of the world's biodiversity as well as carbon.
