# Supplementary material for: A global climate niche for giant trees
Source: Glob Chang Biol. 2018 May 2;24(7):2875–83. doi: 10.1111/gcb.14167 (PMC6033163; doi:10.1111/gcb.14167)
Supplement: Supplementary file 1 [file GCB-24-2875-s001.pdf]

Supporting online material to

Critical Climate conditions for the world's giant trees

Marten Scheffer[1], Chi Xu [2] , Stijn Hantson[3], Milena Holmgren[4], Sietse O. Los [5] and Egbert H. van Nes[1]

1. *Department of Aquatic Ecology and Water Quality Management, Wageningen University, P.O. Box 47, NL-6700 AA, Wageningen, The Netherlands*

2. *School of Life Sciences, Nanjing University, 163 Hankou Road, Nanjing 210023, P.R. China*

3. *Karlsruhe Institute of Technology, Institute of Meteorology and Climate research, Atmospheric Environmental Research, 82467 Garmisch-Partenkirchen, Germany.*

4. *Resource Ecology Group, Wageningen University, P.O. Box 47, NL-6700 AA, Wageningen, The Netherlands*

5. *Department of Geography, Swansea University, Singleton Park, Swansea, SA28PP, UK*

Contents

- Supplementary Figures S1 – S4
- Analysis of relationship to other climate variables with Tables.

Supplementary Figures

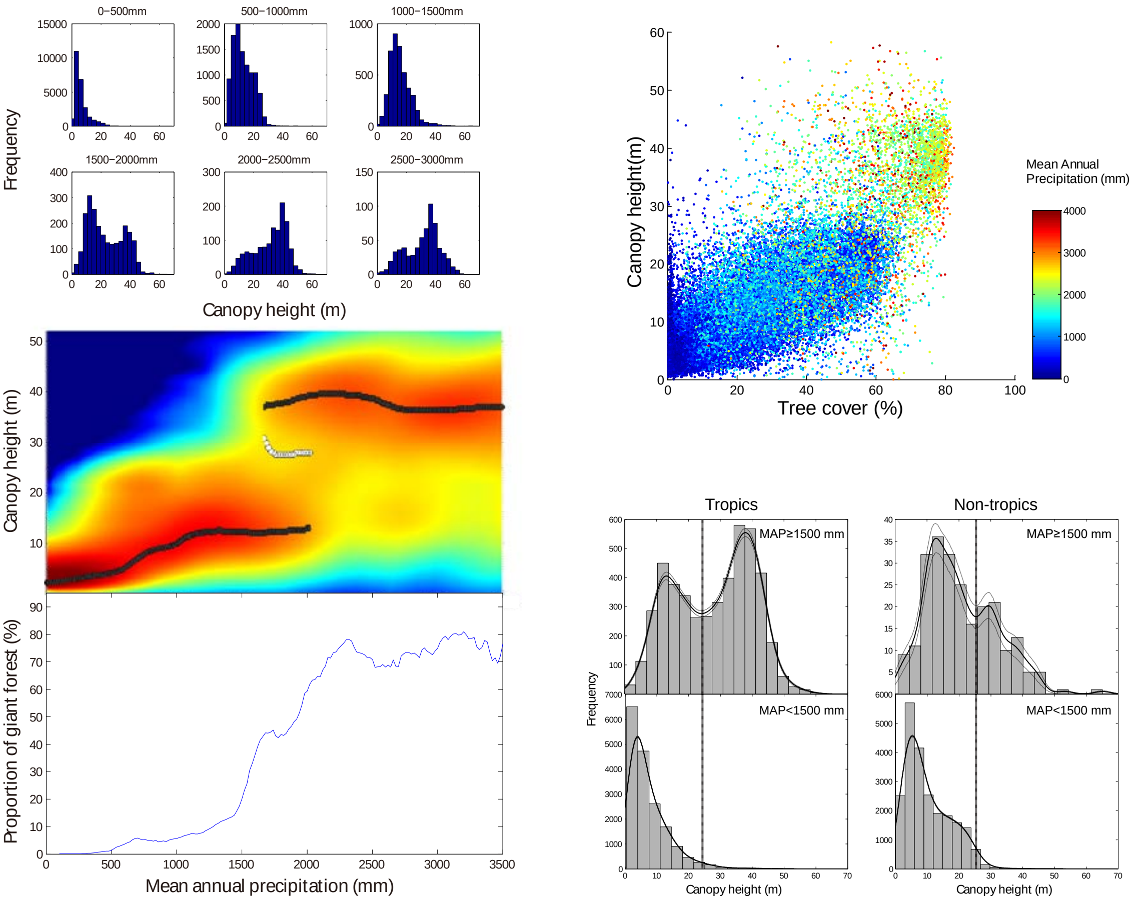

Figure S1. The main results (as presented in Figures 1, 3B and 4 C-F of the main text) but now using the CRU dataset for precipitation.

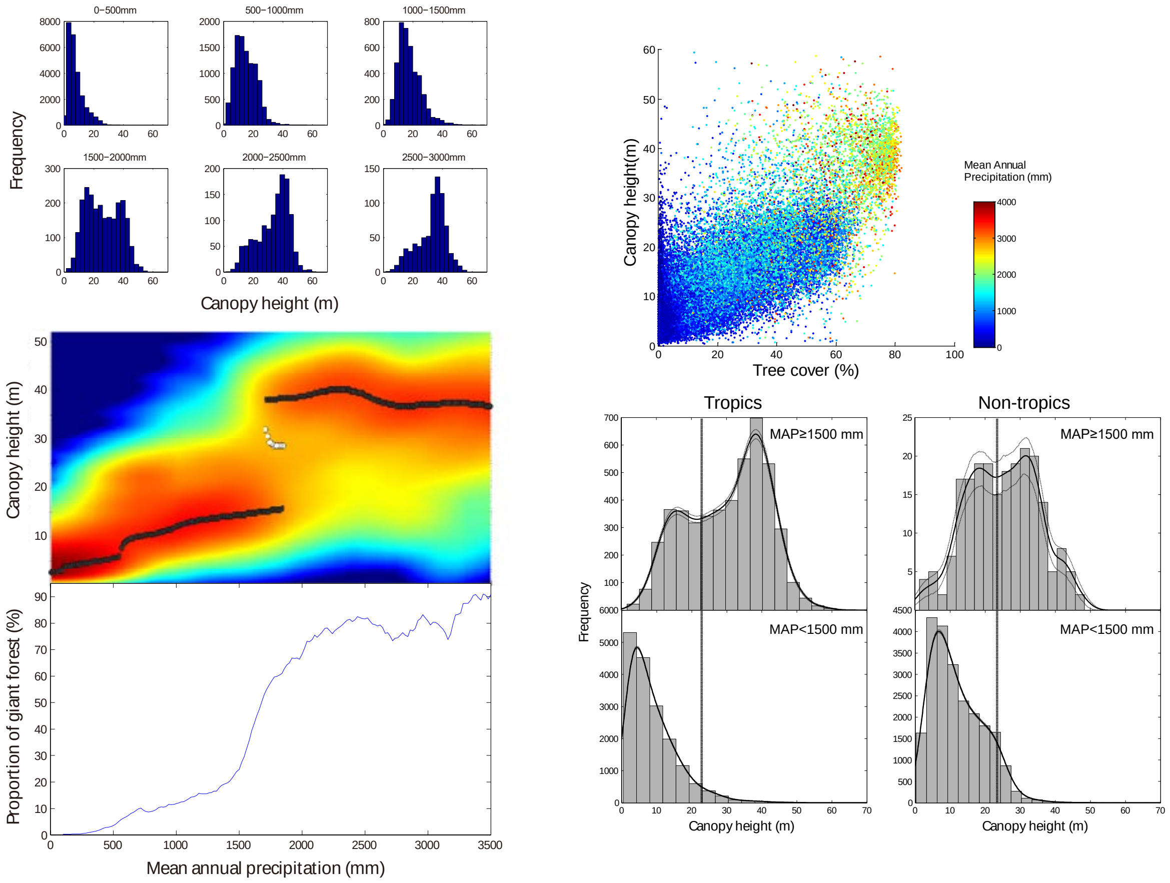

Figure S2. The main results (as presented in Figures 1, 3B and 4 C-F of the main text) but now for an unfiltered set of GLAS data.

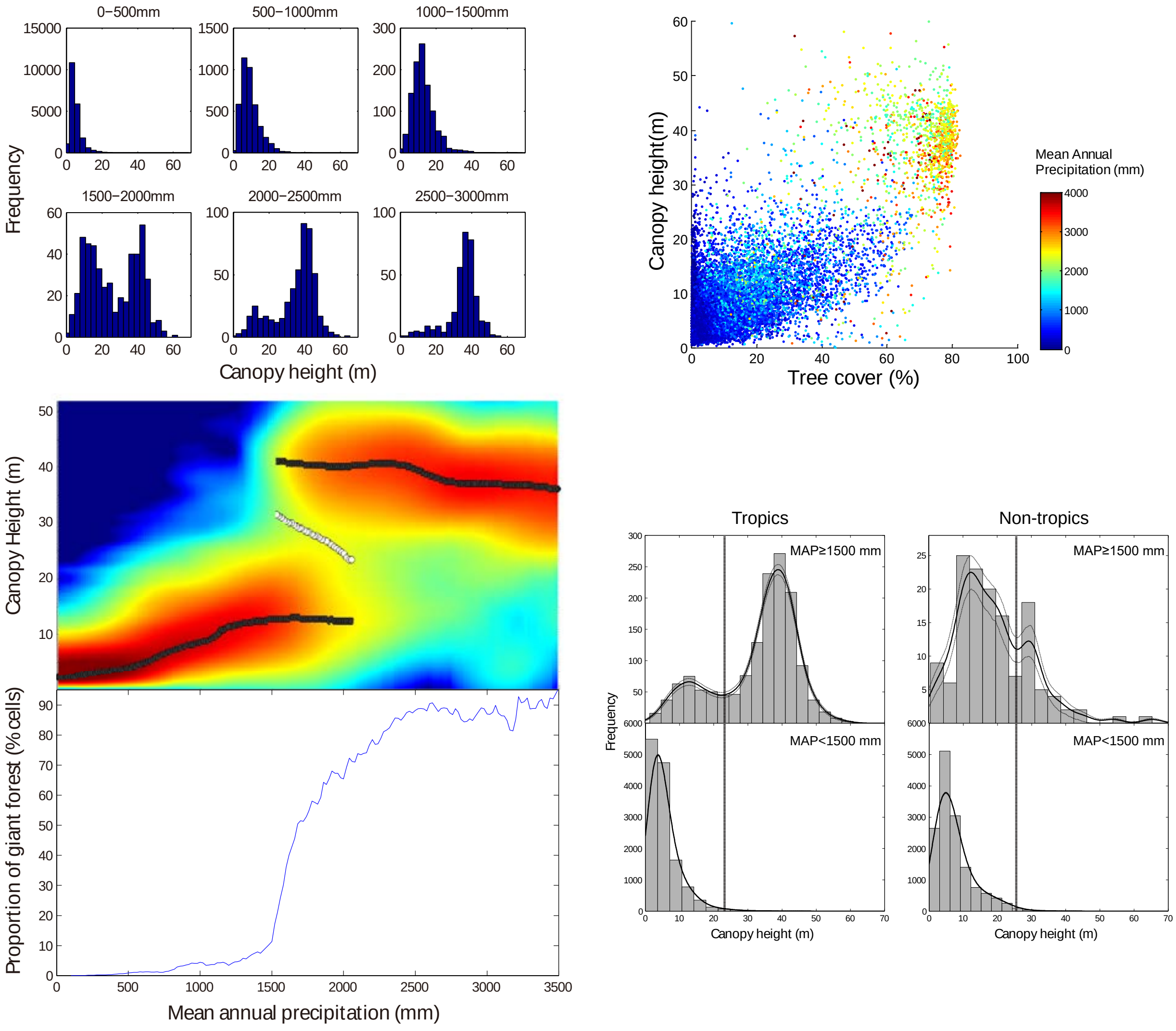

Figure S3. The main results (as presented in Figures 1, 3B and 4 C-F of the main text) but now for a dataset where we removed pixels with signs of harvesting as indicated by a decline of tree cover between 2000 and 2013. To do this, the global forest loss data of compiled by Hansen et al. (2013) were aggregated to 0.5-degree grid cells, and cells with forest loss > 0.5% of the grid cell area were excluded. The robustness of the results suggests that selective harvest may not be a dominant driver of the observed patterns.

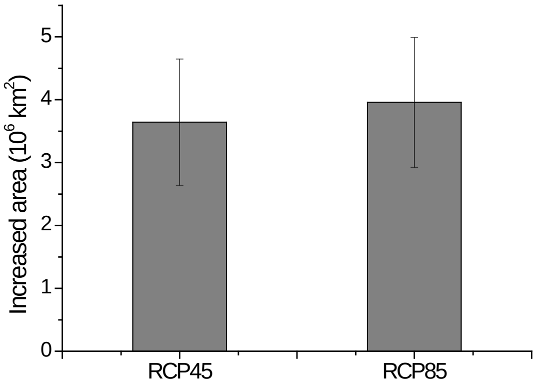

Figure S4. The areas where rainfall exceeds 1500 mm will globally increase by ~4 million km^2. The mean and standard deviation (error bar) are calculated by comparing the current precipitation and the climate projections from global climate models from the Coupled Model Intercomparison Project Phase 5, based on the Representative Concentration Pathway (RCP) of RCP4.5 and RCP8.5. The projected precipitation data are downloaded from the WorldClim website.

Analysis of relationship to other climate variables

The relationship between the presence/absence of giant forests to precipitation and temperature variables is analyzed through spatial generalized linear mixed models with Gaussian spatial structure to deal with the problem of spatial autocorrelation (F Dormann et al., 2007). For the model fitting we extracted random subsets of 2000 (~10%) data points from the tropical and non-tropical region, respectively. The candidate precipitation variables include mean annual precipitation (MAP), Markham's Seasonality Index (MSI), inter-annual variability of precipitation (coefficient of variation of MAP, CV), and extremes (proportion of severely wet and dry years) of precipitation. Severely wet (SPIW) or dry years (SPID) defined as those with yearly precipitation greater than or less than 1.5 times standard deviation of long term MAP were also analyzed but yielded no significant relationships to the occurrence of giant forests. The candidate temperature variables include mean annual temperature (MAT), mean temperature of warmest quarter (TWQ), and mean temperature of coldest quarter (TCQ). WorldClim data of MAP, MAT, TWQ and TCQ data at 1 km resolution were resampled to 0.5×0.5°. Climate Research Unit's (CRU) monthly data (0.5×0.5°) for the period 1961-2001 were used to calculate MSI, CV, SPID and SPIW. Variance inflation factor (VIF) values of the climatic variables are less than 5, indicating no serious multicollinearity. The statistical analysis was performed using R 3.2.1 with the *MASS* package.

Table S1. The spatial generalized linear mixed models accounting for the climate effects on the occurrence of giant forests. The high values of Area Under Curve (AUC) indicate generally good model fitting.

	AUC	Factor	Estimate	Std.
**Tropics (n=2000)**	0.963	MAP	2.190^{***}	0.189
		MSI	-0.927^{***}	0.137
		CV	-0.841^{*}	0.338
**Non-tropics (n=2000)**	0.848	MAP	0.323^{**}	0.098
		CV	-0.956^{***}	0.251
		TWQ	-0.521^{**}	0.199
		TCQ	1.307^{***}	0.254
**Global (n=4000)**	0.938	MAP	1.057^{***}	0.130
		MSI	-0.703^{***}	0.146
		CV	-0.681^{*}	0.310
		TCQ	1.250^{***}	0.356

$^{*}p<0.05$, $^{**}p<0.01$, $^{***}p<0.001$. Standardized coefficients are estimated.

References

F Dormann, C., M McPherson, J., B Araújo, M., Bivand, R., Bolliger, J., Carl, G., . . . Daniel Kissling, W. (2007). Methods to account for spatial autocorrelation in the analysis of species distributional data: a review. *Ecography, 30*(5), 609-628.
